# Denosumab in Cementless Total Hip Arthroplasty: Multivariate Reanalysis of 3D Femoral Stem Migration and the Influence on Outliers

**DOI:** 10.1002/jbm4.10588

**Published:** 2021-12-14

**Authors:** Sami Finnilä, Eliisa Löyttyniemi, Hannu T Aro

**Affiliations:** ^1^ Department of Orthopaedic Surgery and Traumatology Turku University Hospital and University of Turku Turku Finland; ^2^ Unit of Biostatistics, Department of Clinical Medicine University of Turku Turku Finland

**Keywords:** ANTIRESORPTIVES, CLINICAL TRIALS, HIP IMPLANTS, OSTEOARTHRITIS, OSTEOPOROSIS

## Abstract

In cementless total hip arthroplasty, adequate implant stability is necessary for the success of osseointegration and rapid clinical recovery. Postoperative femoral stem migration, measured by radiostereometric analysis (RSA), defines the initial stability achieved during surgical implantation. In a recent trial of 65 postmenopausal women randomized 1:1 denosumab:placebo, denosumab failed to reduce the initial migration of a cementless femoral stem despite the successful prevention of periprosthetic bone loss. The trial applied the current RSA standard, which examined stem migration on an axis‐by‐axis basis and did not consider more complex three‐dimensional (3D) migration. Therefore, we performed a reanalysis of the trial data using a multivariate hierarchical linear mixed model (LMM). As an additional limitation, the data included influential outliers. Women with normal bone mineral density exhibited significantly (*p* = 0.036) less stem subsidence compared with osteopenic and osteoporotic women. Denosumab significantly decreased the variance of stem migration in osteopenic and osteoporotic women. The mean magnitude of 3D stem migration did not differ between denosumab‐treated and placebo‐treated women (*p* = 0.820). After application of a common statistical definition for RSA outlier identification, there were eight (12%) outliers, six in the placebo group and two in the denosumab group (*p* = 0.149). After exclusion of the outliers, the repeated LMM analysis demonstrated a trending difference in 3D stem migration (*p* = 0.086), with a significant difference of *z*‐axis rotation (valgus‐varus tilt) of the femoral stem (*p* = 0.029). The observed effect size was small and without clinically important differences in postoperative recovery. Based on a Monte Carlo simulation with random‐generated 3D migration data, multivariate LMM showed greater statistical power than univariate analyses. The application of hierarchical LMM facilitated the analysis of implant migration as a factual 3D event. The observed trend in the lower number of RSA outliers in denosumab‐treated subjects warrants powered large‐scale trials. © 2021 The Authors. *JBMR Plus* published by Wiley Periodicals LLC on behalf of American Society for Bone and Mineral Research.

## Introduction

In the United States, the registered number of elective primary total hip arthroplasties (THA) was more than 600,000 between 2012 and 2019. Of these procedures, the majority (96%) utilized cementless femoral component fixation.^(^ ^1^ ^)^ The popularity of cementless THA has surpassed that of cemented THA in many other countries.^(^ ^2^, ^3^ ^)^ Stable primary fixation is critical for the success of biological osseointegration; ideally there should not be any early migration at all.^(^ ^4^ ^)^ Clinical femoral stem subsidence may result in failure of implant osseointegration.^(^ ^5^ ^)^ Consequently, there is a tremendous incentive to identify factors that improve primary femoral stem stability.

Postmenopausal women with low bone mineral density (BMD) are prone to initial migration of cementless femoral and acetabular components before osseointegration.^(^ ^6^, ^7^ ^)^ In our recent denosumab trial including postmenopausal women undergoing cementless THA,^(^ ^8^ ^)^ patients were assessed with radiostereometric analysis (RSA), the gold standard for measuring implant migration in vivo.^(^ ^9^, ^10^ ^)^ Contrary to our hypothesis, denosumab failed to reduce femoral stem migration despite the improved periprosthetic bone stock.^(^ ^8^ ^)^ The analysis had two potential limitations. First, the standard RSA examined stem migration on an axis‐by‐axis basis only. The analysis did not consider complex three‐dimensional (3D) migration. Second, the original analysis of the trial data was conducted according to the intention‐to‐treat principle without any exclusion or exploration of outliers. However, influential outliers were identified in further analyses of contributing factors for stem migration.^(^ ^11^, ^12^, ^13^ ^)^ A concern arose over the possibility that the outliers might hide a more subtle treatment effect of denosumab. The selected statistical method, linear mixed modeling (LMM), is also susceptible to outlier‐introduced bias.^(^ ^14^ ^)^ Otherwise, LMM is well suited for the analysis of repeated‐measures multivariate data,^(^ ^15^ ^)^ such as those derived from RSA studies of different femoral stems.^(^ ^16^, ^17^, ^18^ ^)^

Because of these concerns, we decided to perform a multivariate reanalysis of the RSA data from the denosumab trial. The two objectives were: (i) to explore whether hierarchical LMM could be utilized to analyze 3D femoral stem migration, and (ii) to evaluate whether denosumab had an influence on 3D femoral stem migration. We hypothesized that the current standard RSA, involving univariate analysis of axis‐by‐axis migration, might have missed important aspects of the inherent 3D migration of cementless femoral stems in postmenopausal women.

## Materials and Methods

### Study design

This study is a reanalysis of the data from a single‐center, randomized, placebo‐controlled, double‐blinded trial.^(^ ^8^ ^)^ The trial was designed to evaluate the efficacy of denosumab in the prevention of periprosthetic bone loss and in promotion of femoral stem osseointegration (bone bonding) in postmenopausal women with primary hip osteoarthritis undergoing cementless THA (Clinicaltrials.gov NCT01926158). The subjects were randomly assigned to receive a clinical dose of 60 mg every 6 months or placebo for 1 year. All subjects received calcium and vitamin D supplements. The first subcutaneous dose of denosumab or placebo was administered 4 weeks before the surgery. The primary and secondary endpoints were the change in periprosthetic BMD of the proximal femur and migration of the femoral stem at 48 weeks, respectively. The detailed trial protocol and the results were reported previously.^(^ ^8^ ^)^ Further analyses^(^ ^11^, ^12^, ^13^ ^)^ examined the diagnostic accuracy of dual‐energy X‐ray absorptiometry (DXA), quantitative computed tomography (QCT), and pulse‐echo ultrasonometry in predicting stem migration and evaluating the clinical significance of RSA‐measured stem subsidence (≥2 mm). The trial was approved by the Ethics Committee of the Hospital District of South‐West Finland (decisions 105/2012 and 484/2017) and the Finnish Medicines Agency (decision 183/06.00.00/2012, EudraCT 2011–000628‐14). All study participants provided written informed consent before enrollment.

### Screening and trial subjects

According to the inclusion and exclusion criteria,^(^ ^8^ ^)^ the trial included only physically active postmenopausal female patients with normal or close‐to‐normal femur anatomy (Dorr type A or B). Postmenopausal women are likely candidates for prophylactic measures with antiresorptive medication due to the frequency of low BMD,^(^ ^19^, ^20^ ^)^ periprosthetic bone loss,^(^ ^21^ ^)^ and stem migration.^(^ ^7^ ^)^ After cementless THA, women are at increased risk for revision surgery.^(^ ^1^ ^)^ Patients with Dorr type C femurs were excluded because of the increased risk of periprosthetic fractures.^(^ ^22^ ^)^

Screening included hip, lumbar spine, and distal radius BMD evaluation via DXA imaging (Hologic, Discovery A, Hologic Inc., Marlborough, MA, USA). The different regions and bone compartments of the proximal femurs were imaged via QCT^(^ ^23^ ^)^ to measure volumetric BMD (vBMD). All examinations were performed on a single source CT scanner (Somatom Sensation 64, Siemens Healthcare, Forchheim, Germany), utilizing a syngo CT 2009E software version. Helical 120 kVp scans were acquired using bone (sharp) (B70f) or standard (B40F9) kernels for image reconstruction, with a slice thickness and increments of 1 mm and an image matrix of 512 × 512. Serum levels of ionized calcium, 25‐hydroxyvitamin D, and parathyroid hormone were measured to exclude hypocalcemia or vitamin D deficiency before the start of denosumab/placebo administration.

### Surgery and postoperative mobilization

**Fig. 1:**
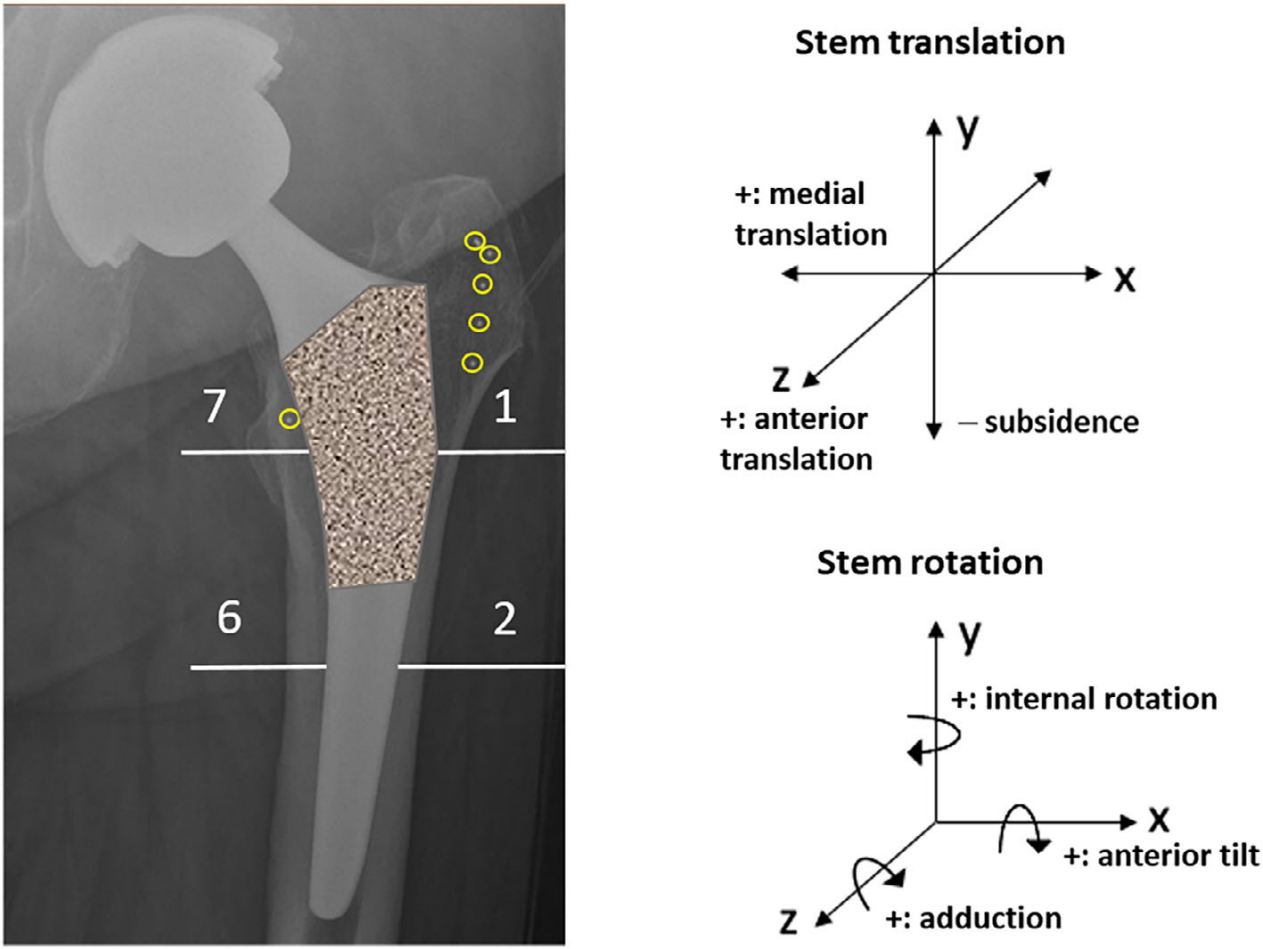
Total hip arthroplasty with parallel‐sided femoral component and tantalum radiostereometric analysis (RSA) bone markers in the trochanteric region (yellow circles). Gruen zones 1–2 and 6–7 of the proximal femur are also marked. To the right is the coordinate system, applied with an external calibration cage during imaging (not shown), for the model‐based RSA analysis.

All subjects underwent standardized cementless THA with implantation of a tapered parallel‐sided single‐wedge femoral stem (Accolade II, Stryker Orthopedics, Mahwah, NJ, USA)^(^ ^24^ ^)^ using the recommended broach‐only technique.^(^ ^25^ ^)^ The stem is the most frequently used femoral component of cementless THA in the United States.^(^ ^1^ ^)^ The investigated stem type requires adequate bone stock and unaltered femoral anatomy.^(^ ^25^ ^)^ During surgery, multiple tantalum RSA beads (1 mm in diameter) were inserted into the trochanteric bone (Fig. 1). Patients were mobilized postoperatively under the supervision of physiotherapists, and unrestricted weight bearing was allowed with the aid of crutches.

### Clinical follow‐up, radiographic assessment, and periprosthetic DXA

The subjects underwent repeated clinical examinations at 12, 22, and 48 weeks. Objective assessment of functional recovery was based on measurements of walking speed and walking activity.^(^ ^8^ ^)^ Concurrently, the Harris hip score (HHS), Western Ontario and McMaster Universities Osteoarthritis Index (WOMAC), and a 36‐item health‐related quality of life survey (Rand‐36 scores) were collected as patient‐reported outcome measures (PROMs).

A computerized method^(^ ^8^ ^)^ was used for the analysis of the canal flare index^(^ ^26^ ^)^ and femoral offset^(^ ^27^ ^)^ from the anteroposterior hip radiograph. As a measure of correct stem size, the ratio of the stem width over the femoral canal width (stem‐to‐canal fill ratio)^(^ ^24^ ^)^ was measured 10 mm above the lesser trochanter (proximal stem), 60 mm below the lesser trochanter (middle stem), and 25 mm above the distal tip of the stem (distal stem) from the postoperative hip radiograph. Radiographic assessment of stem osseointegration was performed at 2 years based on the fixation and stability score criteria.^(^ ^28^ ^)^

DXA measurements for periprosthetic BMDs were performed within 4 days after surgery and repeated at 12, 22, and 48 weeks. The regions of interest, Gruen zones 1 and 2 of the greater trochanter and Gruen zones 6 and 7 of the lesser trochanter (Fig. 1), represent the critical regions of the proximal femur for the primary stability of the implanted femoral stem. The parallel‐sided stem relies on initial press‐fit fixation against the cortical bone in the medial‐lateral plane.^(^ ^29^ ^)^ The proximal coated area of the stem was designed to osseointegrate with the trochanteric region of the proximal femur (Fig. 1).

### Radiostereometric analysis

The trial followed the established RSA guidelines.^(^ ^9^ ^)^ The 3D migration of the femoral stem was measured by model‐based RSA, which utilizes accurate computer‐aided design surface models of each stem size.^(^ ^8^ ^)^ In line with the continuous intra‐laboratory standardization of RSA imaging since 2004,^(^ ^30^ ^)^ the accuracy of model‐based RSA was verified against standard marker‐based RSA in a phantom model before the clinical trial.^(^ ^31^ ^)^ Baseline RSA imaging was performed within 3 days after surgery and repeated at 12, 22, and 48 weeks postoperatively.

For each image, a calibration cage was used to define the global coordinate system.^(^ ^9^ ^)^ The implanted bone markers of the trochanter region acted as a reference rigid body to compute the 3D position of the femoral stem. At each time point, 3D migration was measured for six degrees of freedom as translations along the *x*‐, *y*‐, and *z*‐axes and rotations around the *x*‐, *y*‐, and *z*‐axes (Fig. 1) compared with baseline. As described,^(^ ^8^ ^)^ the analysis was performed using a combined femoral stem‐head model^(^ ^32^ ^)^ (MBRSA software version 3.34; Medis Specials BV, Leiden, The Netherlands). Total translation, total rotation,^(^ ^33^ ^)^ and maximum total point motion (MTPM)^(^ ^9^ ^)^ were computed as surrogate measures of total implant motion and applied as references for the 3D analysis of migration. The stability and sufficient scatter of bone markers were assessed using the mean error of rigid body fitting (ME) and condition number (CN);^(^ ^9^ ^)^ ME >0.35 mm and CN >150 were considered unacceptable. Clinical precision, confirmed with double measurements in 58 patients, was defined as the 95% confidence interval of two repeated measurements.^(^ ^8^ ^)^ The clinical precision for the measurement of stem translation was 140 μm for the *x*‐axis, 110 μm for the *y*‐axis, and 350 μm for the *z*‐axis. The clinical precision for the measurement of stem rotation was 0.50°, 1.04°, and 0.18° for the *x*‐, *y*‐ and *z*‐axis, respectively.^(^ ^8^ ^)^ The clinical precision for the measurement of total translation and rotation were 250 μm and 0.97°, respectively.

### Monte Carlo simulation

To evaluate how multivariate LMM compares to univariate *t* tests in terms of statistical power, a Monte Carlo simulation^(^ ^34^ ^)^ with random‐generated 3D migration data was performed. The distribution of migration data was assumed to be normal. To generate multivariate data, the variance–covariance structure was modeled from the 12‐week results of the RSA data. For each Monte Carlo iteration, a new set of simulated RSA data with two groups (*n* = 30 for each group) was sampled on all six degrees of freedom from two separate populations. As the only distinction between the sampling populations, a mean difference in *y*‐axis translation (subsidence) was introduced so that a univariate *t* test was expected to have a statistical power of 80% at an alpha level of 5%. Before analyzing the data, the migration data in both groups were rotated along a random unit vector on the *x–z*‐axis plane to a defined offset angle from the *y*‐axis. This rotation was introduced to simulate the experimental uncertainty in the alignment of the RSA coordinate system.

Data analysis was then performed at each Monte Carlo iteration using three alternative analytical methods: (i) a two‐sample *t* test on the *y*‐axis translation, (ii) two‐sample *t* tests of both total translation and total rotation, and (iii) a multivariate LMM on all degrees of freedom simultaneously. These Monte Carlo simulation steps were iterated 3000 times for each offset angle. The offset angle ranged from 0° to 90° at five‐degree intervals. For each offset angle, the empirical statistical power for detecting the simulated intergroup difference was recorded and graphed. To demonstrate the potential worst‐case scenario for the multivariate LMM, an otherwise identical Monte Carlo simulation was repeated, where all covariance parameters between the measurement axes were set to zero.

### Statistical analysis

The current LMM analysis was performed with both the original cohort and the cohort without outliers. Seven outliers were found in the two principal directions of postoperative stem migration: distal translation along the *y*‐axis (stem subsidence) and rotation around the *y*‐axis (internal‐external rotation).^(^ ^13^ ^)^ The outliers were identified with a statistical software (IBM SPSS Statistics version 25.0, IBM Corp., Armonk, NY, USA), resulting in the following cut‐off values for the outliers: subsidence >5.44 mm and/or stem rotation >5.52° of internal rotation, or >4.32° of external rotation.^(^ ^13^ ^)^ The outliers were found to be influential in determining the statistical significance between preoperative total hip BMD and *y*‐axis stem translation and rotation.^(^ ^11^ ^)^ The clinical characteristics of the outliers, including age, intertrochanteric vBMD, and cortical bone thickness of the subtrochanteric femur, differed from those of non‐outliers.^(^ ^12^ ^)^ The clinical recovery of the outliers was characterized by large confidence intervals for PROMs, but no failures of osseointegration occurred. The current analysis, focusing on all axes, revealed one additional outlier, which represented a rare case of distal fixation of the femoral component due to a narrow isthmus.^(^ ^35^ ^)^ The subject had a high total hip BMD value (*T*‐score of +2.2) and a complete (100%) canal filling at the middle and distal stems. The subject showed a predominance of stem rotation on *x*‐axis (6.10°, anterior tilt), whereas *y*‐axis rotation and *y*‐axis subsidence were only slightly above the group average values (2.53° and 1.28 mm, respectively).

Comparison of stem migration between denosumab‐treated and placebo‐treated subjects was performed with hierarchical LMM analysis (SAS MIXED Procedure, SAS Institute Inc., Cary, NC, USA).^(^ ^36^ ^)^ The SAS program code is given in Supplemental Table S1. By selecting both the time point and the axis of migration as the repeated measures, the model could include all available RSA data in a single model, allowing the comparison of multivariate (3D) mean migration. The model also allowed the inspection of time‐related changes in the migration patterns between the denosumab and placebo groups over the entire 1‐year trial period. The analysis accounted for the varying scale, the differences in measurement noise, autocorrelation over all axes of measurement, and time points. This was done by estimating the relevant covariance–variance matrices based on the available data (ie, the covariance–variance matrices were specified as unstructured for both repeated measures). The treatment group was included as the main fixed effect, and all relevant interactions of the treatment groups, time points, and axes of migration were included. The model was subsequently simplified by dropping non‐significant interactions from the model.

The model residuals were assessed for an acceptable normal distribution through visual inspection of the Q‐Q‐plots. The validity of the chosen model was confirmed by plotting the studentized model residuals against the predicted values. The significance testing of model effects was facilitated with the Kenward–Roger approximation for denominator degrees of freedom. As LMM allows for case‐wise missing values, all available data points were used to build the model without the need for imputation. Three RSA observations were missing (2.1%) due to technical reasons: one at the 12‐week visit and two at the 24‐ and 48‐week visits.

The level of statistical significance was set at *p* < 0.05. Analyses were performed using SAS System version 9.4 (SAS Institute).

The Monte Carlo simulation of generated data was performed using R (version 4.0.3; R Foundation for Statistical Computing, Vienna, Austria) with lme4 (version 1.1‐26), lmerTest (version 3.1‐3), and pbkrtest (version 0.5‐0.1) packages to enable LMM along with the Kenward–Roger approximation. The MASS package (version 7.3‐53) was used to generate random multivariate normal data. The rotations (version 1.6.1) and varbvs (version 2.5‐16) packages were used to generate the random rotation matrices. Welch's two‐sample *t* test was used for the univariate analyses.

## Results

### Original cohort

**Fig. 2:**
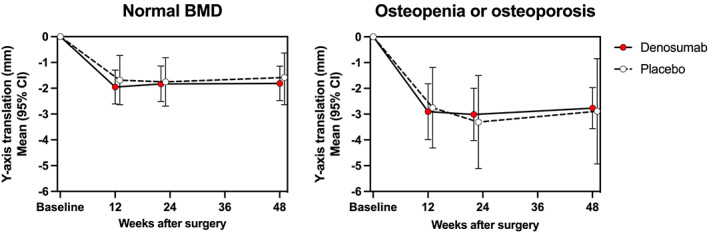
Femoral stem subsidence (*y*‐axis translation) as a function of postoperative time in the denosumab and placebo groups (the original cohort): mean changes from baseline (and 95% confidence interval) were calculated. The difference between subjects with normal or low (osteopenia or osteoporosis) bone mineral density (BMD) was statistically significant (*p* = 0.036). In subjects with osteopenia or osteoporosis, the variance of subsidence differed significantly (*p* = 0.006) between denosumab‐treated and placebo‐treated subjects at 48 weeks.

**Fig. 3:**
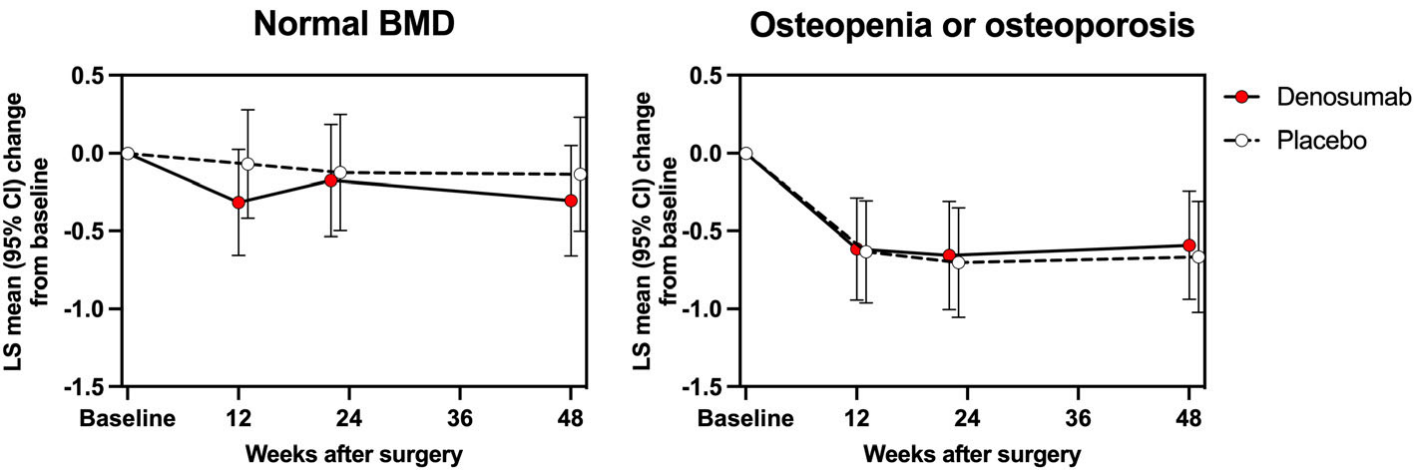
Three‐dimensional femoral stem migration as a function of postoperative time in the denosumab and placebo groups (the original cohort): least‐squares (LS) mean changes from baseline (and 95% confidence interval) were calculated for 3D stem migration using hierarchical linear mixed model (LMM) analysis.

The analysis of the original cohort (*n* = 65) revealed a close relationship between preoperative BMD, stem migration, and denosumab treatment. Women with normal BMD exhibited significantly (*p* = 0.036) less stem subsidence compared with osteopenic and osteoporotic women (Fig. 2). Denosumab reduced the variance of stem migration among osteopenic or osteoporotic subjects, measured by subsidence (*p* = 0.006, Fig. 2), total translation (*p* = 0.001), total rotation (*p* = 0.041) and maximum total point motion (*p* = 0.001) at 48 weeks (Levene's test for equality of variances), but the mean values of stem migration did not differ between the denosumab and placebo groups. The hierarchical LMM reanalysis of the original cohort revealed no significant difference of 3D stem migration between the denosumab and placebo groups (*p* = 0.814), but there was a trend (*p* = 0.181) for less 3D stem migration in subjects with normal BMD compared with osteopenic and osteoporotic subjects (Fig. 3).

### Cohort without outliers

Of the eight excluded outliers, two were denosumab‐treated (2/33, 6%) and six were placebo‐treated (6/32, 19%) (Fisher exact test, *p* = 0.149). The baseline characteristics of the cohort without outliers (*n* = 57) were balanced between the denosumab and placebo groups (Tables 1 and 2). Denosumab increased periprosthetic BMD above the baseline in the greater (Gruen zone 1) and lesser trochanteric regions (Gruen zone 6) (Table 3). The treatment response became evident rapidly (within 12 weeks) in Gruen zone 1, reflecting the local predominance of the trabecular bone. Placebo‐treated subjects exhibited periprosthetic bone loss in all proximal Gruen zones (Table 3).

**Table 1:** Baseline Patient Characteristics

	Denosumab (*n* = 31)	Placebo (*n* = 26)	*p* Value^a^
Age at consent (years)			
Mean ± SD (range)	68.6 ± 5.0 (61–79)	69.1 ± 6.1 (60–84)	0.720
BMI, mean ± SD (kg/m^2^)	27.8 ± 5.5	28.2 ± 3.8	0.731
ASA			
Class I–II, *n* (%)	17 (55)	17 (65)	0.600
Class III, *n* (%)	14 (45)	9 (35)	
History of low‐energy fractures, *n* (%)			
Yes	8 (26)	7 (27)	1.000
No	23 (74)	19 (73)	
25‐hydroxyvitamin D, mean ± SD (nmol/L)	97.7 ± 28.1	95.7 ± 30.1	0.792
WHO classification of aBMD, *n* (%)^b^			
Normal aBMD	16 (52)	13 (50)	0.389
Osteopenia	13 (42)	13 (50)	
Osteoporosis	2 (6)	0 (0)	
Bone dimensions			
Radius cortical thickness, mean ± SD (mm)^c^	2.5 ± 0.7	2.5 ± 0.9	0.939
Canal flare index, mean ± SD	3.8 ± 0.7	3.8 ± 0.6	0.945
Femoral stem size, mean (range)	3.2 (1–6)	3.3 (2–5)	0.679
Femoral offset			
Preoperative, mean ± SD (mm)	38.0 ± 5.2	38.0 ± 4.5	0.960
Postoperative, mean ± SD (mm)	37.8 ± 5.3	37.7 ± 5.1	0.920
Stem‐to‐canal fill ratio			
Proximal stem (%)	98.1 ± 2.3	97.2 ± 2.5	0.166
Middle stem (%)	86.4 ± 7.0	85.5 ± 10.0	0.684
Distal stem (%)	84.8 ± 7.9	84.6 ± 10.5	0.938

BMI = body mass index; ASA = American Society of Anesthesiologists; WHO = World Health Organization; aBMD = areal bone mineral density.

^a^ The *p* values are from two independent samples *t* test for normally distributed variables. For categorical variables, *p* values are from chi‐square test or Fisher exact test.

^b^ Based on *T*‐scores of the lumbar spine and the hips.

^c^ Measured by pulse‐echo ultrasound.

**Table 2:** Baseline Characterization by Multisite DXA, Proximal Femur QCT, and Periprosthetic DXA

	Denosumab	Placebo	*p* Value^a^
**Multisite DXA**	*n* = 31	*n* = 26	
Total hip BMD (g/cm^2^)^b^	0.917 ± 0.164	0.931 ± 0.117	0.713
Femoral neck BMD (g/cm^2^)^b^	0.819 ± 0.150	0.859 ± 0.118	0.274
Lumbar spine BMD (g/cm^2^)^b^	1.018 ± 0.191	0.983 ± 0.148	0.439
Distal radius BMD (g/cm^2^)^b^	0.646 ± 0.074	0.671 ± 0.064	0.178
**Proximal femur QCT**	*n* = 27	*n* = 21	
**Total hip**			
Integral vBMD (mg/cm^3^)^b^	304.7 ± 59.4	313.6 ± 41.5	0.563
Cortical bone vBMD (mg/cm^3^)^b^	683.8 ± 69.1	703.9 ± 80.0	0.356
Trabecular bone vBMD (mg/cm^3^)^b^	121.4 ± 47.6	133.7 ± 29.9	0.309
Cortical bone thickness (mm)^b^	2.08 ± 0.31	1.96 ± 0.36	0.230
**Femoral neck**			
Integral vBMD (mg/cm^3^)^b^	348.3 ± 75.0	364.8 ± 46.8	0.384
Cortical bone vBMD (mg/cm^3^)^b^	661.3 ± 83.5	672.8 ± 67.7	0.609
Trabecular bone vBMD (mg/cm^3^)^b^	162.9 ± 64.0	185.0 ± 41.8	0.177
Cortical bone thickness (mm)^b^	1.98 ± 0.31	1.92 ± 0.34	0.519
**Intertrochanteric region**			
Integral vBMD (mg/cm^3^)^b^	335.2 ± 71.4	343.9 ± 52.0	0.642
Cortical bone vBMD (mg/cm^3^)^b^	832.6 ± 84.5	859.4 ± 95.0	0.306
Trabecular bone vBMD (mg/cm^3^)^b^	113.7 ± 47.3	128.7 ± 31.3	0.217
Cortical bone thickness (mm)^b^	2.50 ± 0.41	2.38 ± 0.52	0.362
**Periprosthetic DXA**	*n* = 31	*n* = 26	
Gruen 1 BMD (g/cm^2^)^b^	0.763 ± 0.134	0.740 ± 0.125	0.521
Gruen 2 BMD (g/cm^2^)^b^	1.475 ± 0.266	1.479 ± 0.150	0.951
Gruen 6 BMD (g/cm^2^)^b^	1.354 ± 0.253	1.363 ± 0.200	0.880
Gruen 7 BMD (g/cm^2^)^b^	1.077 ± 0.224	1.091 ± 0.204	0.810

DXA = dual‐energy X‐ray absorptiometry; QCT = quantitative computed tomography; BMD = bone mineral density; vBMD = volumetric bone mineral density.

^a^ The *p* values are from two independent‐samples *t* test.

^b^ The values are mean ± SD.

**Table 3:** Postoperative Changes of Periprosthetic Bone Mineral Density

	Denosumab (*n* = 31)	Placebo (*n* = 26)	Mean difference (95% CI)
**The greater trochanteric region**		
**Gruen zone 1**			
12 weeks^a^	4.75 (2.01 to 7.49)	−3.87 (−7.22 to −0.51)	8.62 (4.43 to 12.80)
22 weeks^a^	3.42 (0.29 to 6.54)	−4.99 (−8.48 to −1.50)	8.41 (3.84 to 12.97)
48 weeks^a^	7.65 (3.00 to 12.30)	−4.77 (−9.18 to −0.36)	12.4 (6.04 to 18.79)
**Gruen zone 2**			
12 weeks^a^	−1.14 (−3.95 to 1.68)	−6.50 (−9.22 to −3.78)	5.36 (1.48 to 9.25)
22 weeks^a^	−1.59 (−4.42 to 1.25)	−6.60 (−8.83 to −4.37)	5.02 (1.39 to 8.65)
48 weeks^a^	0.37 (−1.71 to 2.46)	−3.34 (−5.94 to −0.73)	3.71 (0.49 to 6.92)
**The lesser trochanteric region**		
**Gruen zone 6**			
12 weeks^a^	3.43 (−0.17 to 7.03)	−3.95 (−6.68 to −1.22)	7.38 (2.78 to 11.99)
22 weeks^a^	3.54 (0.81 to 6.29)	−3.12 (−6.23 to −0.02)	6.67 (2.64 to 10.70)
48 weeks^a^	4.92 (1.43 to 8.41)	−1.71 (−5.01 to 1.59)	6.64 (1.86 to 11.42)
**Gruen zone 7**			
12 weeks^a^	−2.31 (−6.44 to 1.82)	−13.56 (−17.38 to −9.73)	11.24 (5.63 to 16.86)
22 weeks^a^	−6.98 (−10.44 to −3.53)	−16.54 (−20.09 to −13.00)	9.56 (4.70 to 14.42)
48 weeks^a^	−4.71 (−7.94 to −1.48)	−16.82 (−20.92 to −12.72)	12.11 (7.09 to 17.14)

^a^ Values are expressed as percent change from baseline (95% confidence interval [CI]).

**Fig. 4:**
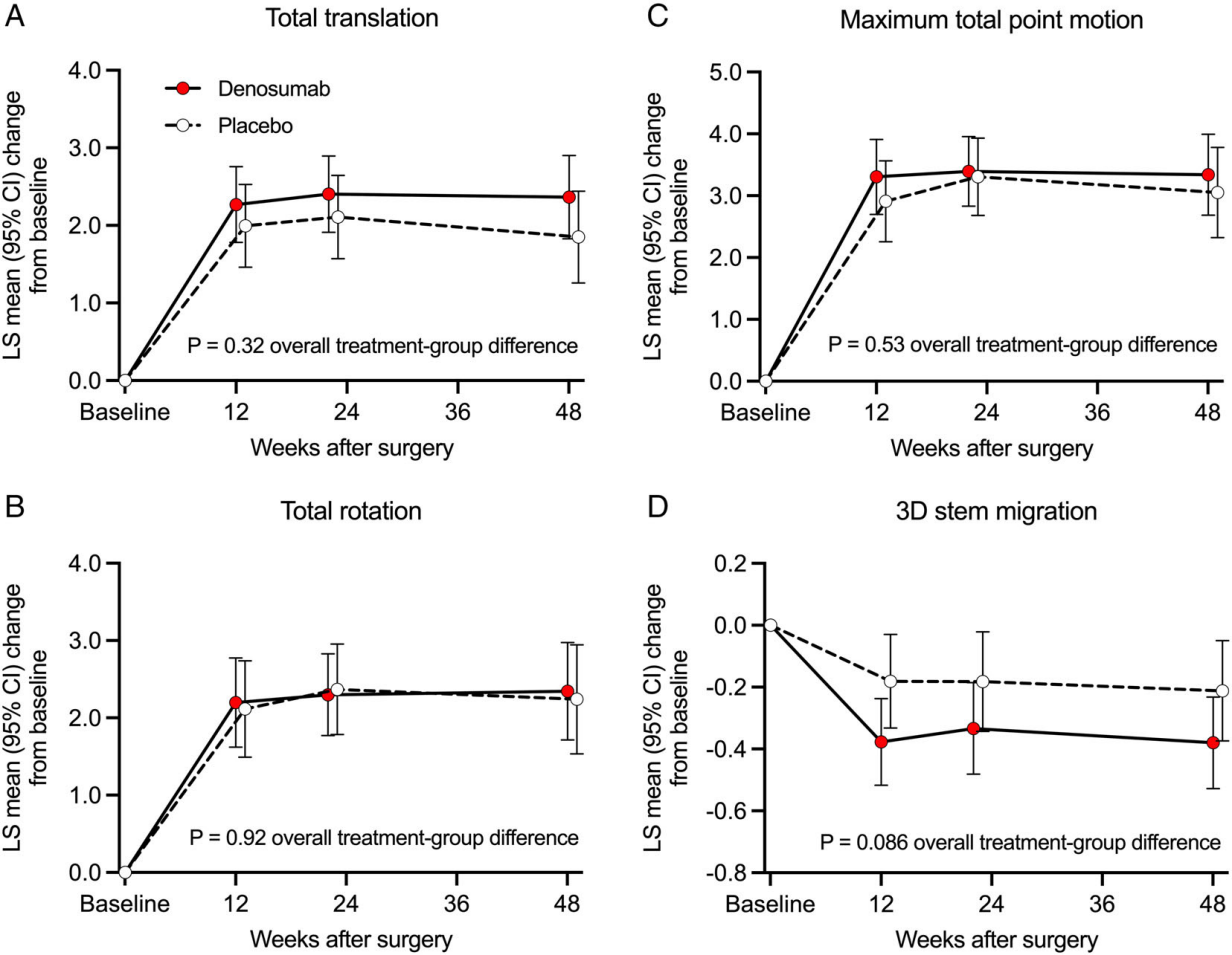
Evaluation of total implant migration as a function of postoperative time in the denosumab and placebo groups (the cohort without outliers): least‐squares (LS) mean changes from baseline (and 95% confidence interval) were calculated for the femoral stem migration as total translation vector lengths (*A*), total rotation vector lengths (*B*), maximum total point motion (*C*), and 3D stem migration measured by hierarchical linear mixed model (LMM) analysis (*D*). There was a trend (*p* = 0.086) for a difference of 3D stem migration between denosumab‐treated and placebo‐treated subjects.

**Fig. 5:**
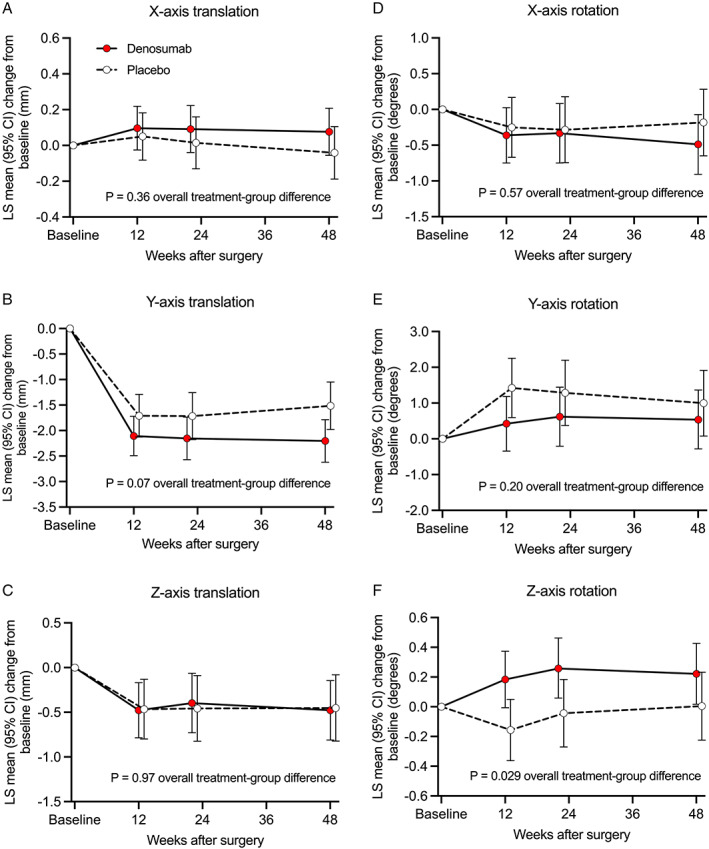
Comparison of femoral stem migration between the denosumab and placebo groups on an axis‐by‐axis basis (the cohort without outliers): least‐squares (LS) mean changes from baseline (and 95% confidence interval) were calculated for translation along *x*‐axis (*A*), *y*‐axis (*B*), and *z*‐axis (*C*) and in rotation around *x*‐axis (*D*), *y*‐axis (*E*), and *z*‐axis (*F*). The *z*‐axis rotation showed a significant (*p* = 0.029) difference between denosumab‐treated and placebo‐treated subjects.

**Fig. 6:**
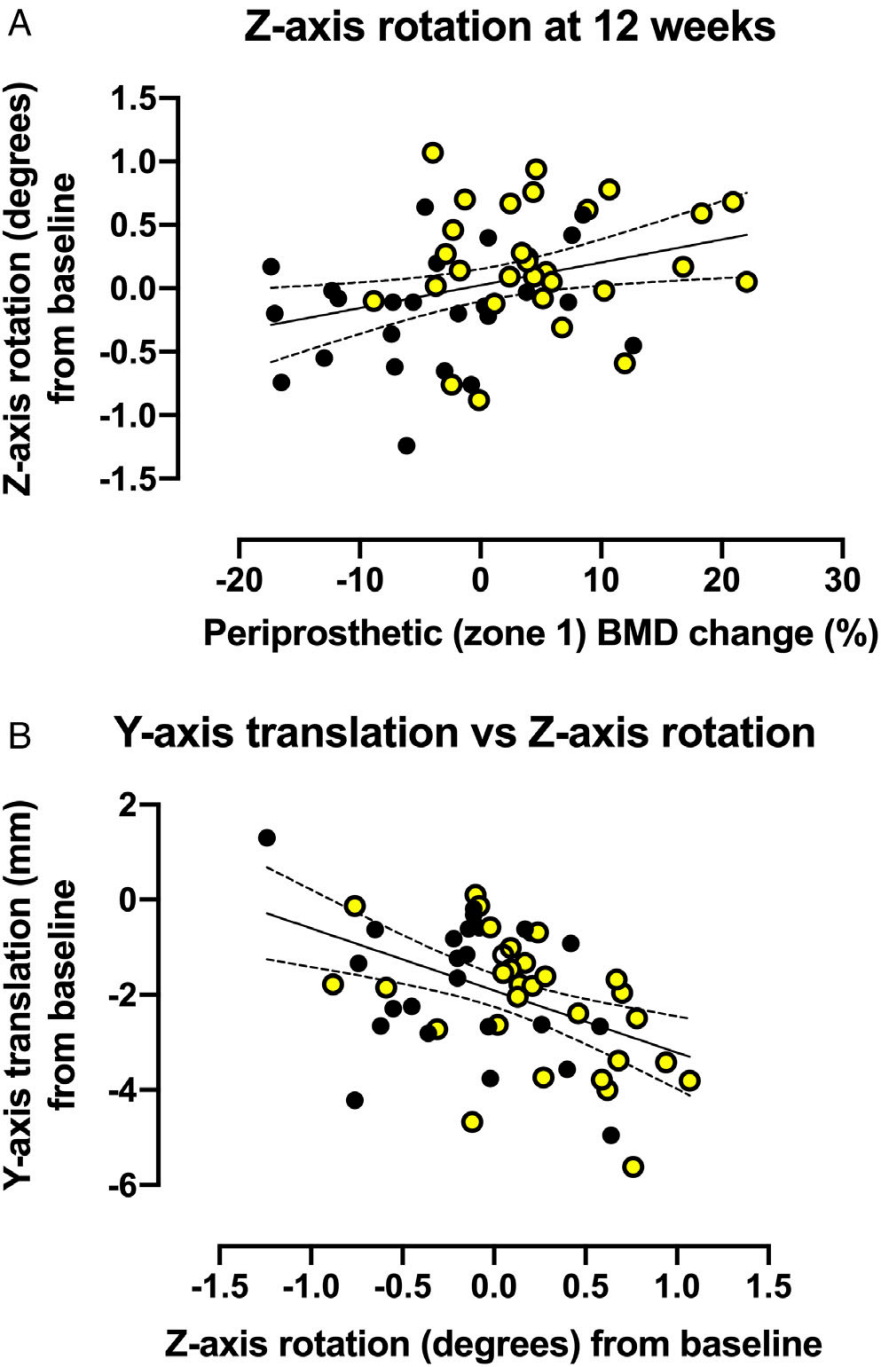
Linear regression analyses of the associations between *z*‐axis rotation and the changes of periprosthetic bone mineral density (BMD; Gruen zone 1) (*A*) and *y*‐axis translation (*B*) during the first 12 weeks after surgery (Pearson correlation coefficients *r* = 0.325 [*p* = 0.016] and *r* = −0.449 [*p* = 0.001], respectively). Yellow dots represent individual values of the denosumab group and black dots those of the placebo group.

When the LMM analysis was repeated with the exclusion of the outliers, a trend was observed in 3D stem migration during the 48‐week postoperative period (*p* = 0.086) (Fig. 4*D*). An inspection of LMM effect slices showed a significant difference in *z*‐axis rotation (valgus‐varus) between denosumab‐treated and placebo‐treated subjects (*p* = 0.029) (Fig. 5*F*). The direction of *z*‐axis rotation was into a valgus position in the denosumab group. The estimated difference (least squares mean [LSM] in *z*‐axis rotation was 0.30°; 95% confidence interval [CI] 0.03 to 0.56). The *z*‐axis rotation was observed predominantly during the first 12 weeks (Fig. 5*F*). In exploratory post hoc analyses, *z*‐axis rotation was associated with concurrent changes in periprosthetic BMD in Gruen zone 1 (Fig. 6*A*) and *y*‐axis translation (subsidence) (Fig. 6*B*).

The two groups showed no significant differences in clinically important subsidence (≥2 mm), which occurred in 16 subjects (52%) in the denosumab group and in 12 subjects (46%) in the placebo group (*p* = 0.792, Fisher exact test). The number of subjects achieving the minimum clinically important improvement (MCII) of PROMs did not differ between the two groups. In both groups, 77% of the subjects achieved the MCII of HHS score (≥18 points). In the denosumab group, 37% of the subjects achieved the MCII of postoperative walking speed (≥0.32 m/s) compared with 44% of the subjects in the placebo group (*p* = 0.778, Fisher exact test).

Based on the review of electronic medical records, no revision of any implant component has been performed at the minimum follow‐up of 5 years (range 5.0 to 6.9 years). There were two deaths, for unrelated reasons.

### Monte Carlo simulation

**Fig. 7:**
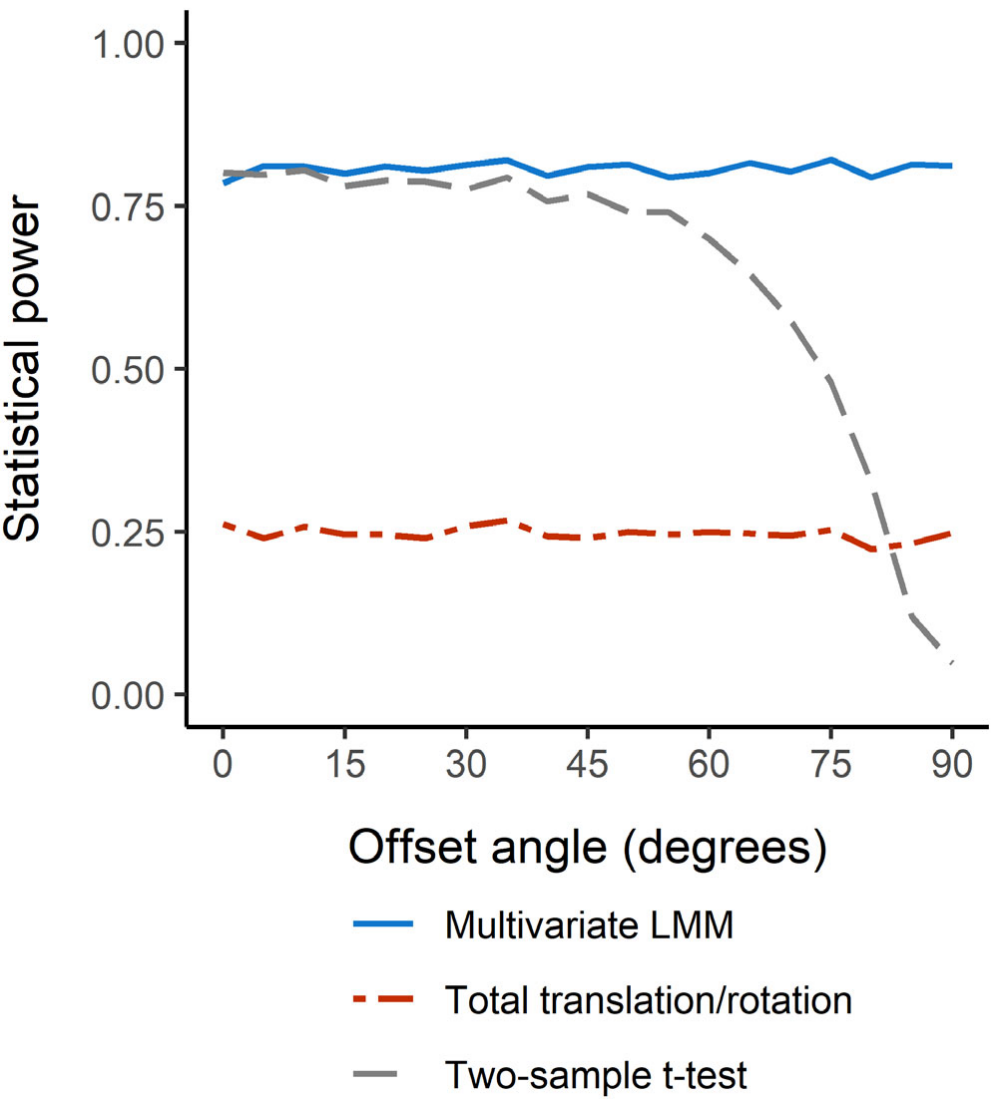
Comparison of the statistical power in simulated radiostereometric analysis (RSA) data of two hypothetical groups for three methods: a multivariate linear mixed model (LMM), a univariate *t* test done on *y*‐axis translation alone, and *t* tests of both total translation and total rotation. The statistical power is graphed as a function of rotating the migration data to an offset angle from the axis with the simulated intergroup difference (*y*‐axis translation) before analysis. Each data point corresponds to 3000 iterations of a Monte Carlo simulation.

Multivariate LMM had greater statistical power over the combined range of simulated offset angles compared with the univariate analyses to detect migration (Fig. 7); (81% versus 64%, respectively). The statistical power of the *t* test was considerably reduced beyond approximately 50°. Total translation and total rotation, analyzed with two separate *t* tests, had an average statistical power of 25%. In the worst‐case scenario of no correlation between any of the measurement axes, multivariate LMM still had a statistical power of 48%.

## Discussion

In total hip arthroplasty, RSA provides a means to accurately measure postoperative femoral stem migration. A limitation of the current RSA standard is the analysis of migration on an axis‐by‐axis basis.^(^ ^9^ ^)^ We explored the suitability of hierarchical LMM in the analysis of factual 3D implant migration. The reanalysis of the original cohort revealed no intergroup difference of 3D stem migration between denosumab‐treated and placebo‐treated patients. The LMM analysis of the cohort without outliers revealed a significant difference between the two groups in *z*‐axis rotation (valgus‐varus tilt) of the femoral stem. The scalar difference in *z*‐axis rotation was small, but this difference was along the long axis of the stem, whereby even small rotations translate to modest movements at either end of the implant. The two groups showed, however, no clinically meaningful differences in postoperative recovery.

Using the 3D modeling strategy, multivariate LMM has theoretical advantages over analysis of individual axes, separately. First, the alignment of the RSA coordinate system, based on anatomical landmarks and directions, is quite arbitrary from the viewpoint of 3D implant migration. Even if most of the clinically meaningful information in the data were aligned with any RSA axis, it is an entirely theoretical notion that all relevant migration were contained within a single axis at any one time. Second, the variance of an RSA sample is also multivariate with confidence intervals of mean estimates resembling a multidimensional ellipsoid. As an extreme example, it is possible to have RSA data with no discernible differences in univariate terms while simultaneously having a categorical multivariate intergroup difference. Third, the use of a single statistical model also effectively mitigates the issue of multiplicity. RSA trials are inherently susceptible to issues related to multiplicity. The multidimensional main outcome variable, 3D migration, combined with a typical longitudinal study setting with multiple time points, leads to a large number of potential comparisons.

LMM is not the only analytical method applicable to multivariate longitudinal data analysis.^(^ ^37^ ^)^ However, it is perhaps one of the most flexible in terms of modeling strategies, has good availability in the form of established software packages, and has been extensively studied in the analysis of real‐valued longitudinal data.^(^ ^37^, ^38^ ^)^ Another advantage of LMM is that it accommodates missing observations over the repeated factors, a common occurrence in RSA data, and it has even been suggested to be the method of choice for this type of data.^(^ ^39^, ^40^, ^41^ ^)^ However, as applied in the present study, LMM is best equipped for detecting differences and changes in *mean* migration. This can lead to misinterpretation of data if the migration only differs in scale or variance, while the mean migration remains equal between the groups.^(^ ^42^ ^)^ This challenge was encountered in our LMM analysis of the original cohort.

In the Monte Carlo simulation, the multivariate nature of the underlying 3D implant migration was represented by the variance–covariance structure modeled from the trial data. As a result, multivariate LMM maintained similar or better statistical power than a univariate *t* test for the detection of significant migration while providing a more comprehensive hypothesis testing of the data on all axes simultaneously. Even in the hypothetical worst‐case scenario of zero covariance between the degrees of freedom in the data, multivariate LMM still had a lower bound statistical power, far better than that achieved with the analysis of total translation and rotation.

Surrogate measures for 3D implant motion, such as total translation and rotation vector lengths and MTPM, are inherently limited by the loss of freedom degrees when calculating them.^(^ ^9^, ^42^ ^)^ This deficiency was also evident in the comparative Monte Carlo simulation. Still, the use of these surrogates is justified when there is strong precedence for their use (eg, MTPM in trials of total knee arthroplasties).^(^ ^43^ ^)^

Stem subsidence of ≥2 mm, carrying the risk of slower functional recovery, is associated with low intertrochanteric vBMD in postmenopausal women with high levels of bone resorption and bone formation serum markers.^(^ ^12^ ^)^ As a highly potent antiresorptive drug, denosumab improves the structure of the proximal femur in postmenopausal women with osteoporosis^(^ ^44^, ^45^ ^)^ and has a clear impact on the maintenance of periprosthetic bone stock. Therefore, it was reasonable to expect that denosumab would decrease the rate of stem subsidence by ≥2 mm; however, this did not happen in our study, probably attributable to the late denosumab administration before surgery.

The outliers had a significant effect on the outcome of the analysis. In our trial, RSA outliers with major stem migration before osseointegration were clinically distinguishable in terms of demographics and bone quality.^(^ ^12^ ^)^ Thus, the supplementary analysis without the outliers was in our view justified. With the exclusion of the outliers, the effects of denosumab could be more closely examined in absence of the measurement noise introduced by the outliers. Nevertheless, the effect of denosumab on the outliers is equally interesting. If denosumab could reduce the number of the RSA outliers, this would represent a clinically important finding. We observed a trend in the lower number of RSA outliers in denosumab‐treated subjects, but the trial was not powered to examine the efficacy of denosumab to prevent excessive migration (ie, outliers). Using the observed rate of RSA outliers in the current trial, the required group size was 105 (two‐sided α = 0.05, β = 0.80). Ultimately, the debate on how to optimally treat outliers in RSA data analysis is ongoing.^(^ ^46^, ^47^ ^)^ For this reason, we presented the LMM reanalysis both with and without the outliers.

On purpose, our study included only postmenopausal women. The factors causing stem migration appear to be different in men.^(^ ^48^ ^)^ As a limitation, the results are applicable only to the investigated implant model. These facts decrease the generalizability and transferability of our results. The main weakness of our study was the unplanned post hoc nature of the reanalysis. Therefore, our findings should be considered exploratory. In addition, the LMM analysis of 3D migration showed only a trend, which we nevertheless interpreted as warranting a closer axis‐by‐axis examination of the model results. Finally, we adopted the common definition (1.5× interquartile range above the upper quartile or below the lower quartile) for RSA outlier identification. In LMM analyses, an option would be to consider using proper model influence diagnostics (eg, restricted maximum likelihood distance) for the detection of influential observations and multivariate (3D) outliers.^(^ ^49^ ^)^

In conclusion, the application of hierarchical LMM facilitated the analysis of implant migration as a factual 3D event. It was found to be a valuable analytical method for improving the robustness and power of statistical inference from clinical RSA data. The best strategy for handling clinical and RSA outliers remains an open question. In any case, it seems relevant that randomized RSA trials of cementless total hip arthroplasties are powered to examine the efficacy of an intervention in the prevention of outliers.

## Disclosures

HTA has received institutional research grants (Amgen) and a consultation fee (UCB Biopharma Sprl). The other authors declare that they have no conflicts of interest.

### Peer Review

The peer review history for this article is available at https://publons.com/publon/10.1002/jbm4.10588.

## Supporting information


**Appendix S1.** Supporting Information.

## Data Availability

The data that support the findings of this study are available on request from the corresponding author. The data are not publicly available due to privacy or ethical restrictions.
