# Supplementary material for: Denosumab in Cementless Total Hip Arthroplasty: Multivariate Reanalysis of 3D Femoral Stem Migration and the Influence on Outliers
Source: JBMR Plus. 2021 Dec 14;6(2):e10588. doi: 10.1002/jbm4.10588 (PMC8861983; doi:10.1002/jbm4.10588)
Supplement: Supplementary file 1 — Appendix S1. Supporting Information. [file JBM4-6-e10588-s001.docx]

**Supplementary data – SAS® MIXED Procedure**

**PROC** **MIXED** DATA=XXX covtest plot=studentpanel;

CLASS id group migration_axis time;

MODEL response = migration_axis group*migration_axis time*migration_axis group*time*migration_axis / residual NOINT DDFM=KENWARDROGER2;

REPEATED migration_axis time / SUBJECT=id TYPE=UN@UN;

LSMEANS group*migration_axis time*migration_axis group*time*migration_axis /diff cl;

**run**;

| **Variable** | **Description** |
| --- | --- |
| group | Patient group: e.g., placebo vs active treatment. |
| migration_axis | Axis of migration. Rotation and translation axes are treated as their own separate axes. This allows including all 6 degrees of migration into the same statistical model. |
| time | The follow-up timepoint of the RSA examination. |

For a more thorough explanation of the used script, please, refer to:

1. SAS Institute Inc. 2018. SAS/STAT® 15.1 User’s Guide. Cary, NC: SAS Institute Inc.
